# Glioblastoma Stem Cells at the Nexus of Tumor Heterogeneity, Immune Evasion, and Therapeutic Resistance

**DOI:** 10.3390/cells14080562

**Published:** 2025-04-09

**Authors:** Justin Tang, Md Al Amin, Jian L. Campian

**Affiliations:** 1 Department of Biomedical Science, University of Guelph, Guelph, ON N1G 2W1, Canada; 2 Department of Oncology, Mayo Clinic, Rochester, MN 55905, USA; amin.mdal@mayo.edu (M.A.A.); campian.jian@mayo.edu (J.L.C.)

**Keywords:** glioblastoma, glioblastoma stem cells, tumor heterogeneity, immune evasion, therapeutic resistance, self-renewal, DNA repair, metabolic adaptations, perivascular niche, hypoxic microenvironment, notch signaling, Wnt/β-catenin pathway, Hedgehog pathway, STAT3-PARN axis, TFPI2, HML-2, immunosuppression, CAR T-cell therapy, patient-derived organoids, single-cell omics

## Abstract

Glioblastoma (GBM) is an exceedingly aggressive primary brain tumor defined by rapid growth, extensive infiltration, and resistance to standard therapies. A central factor driving these malignancies is the subpopulation of glioblastoma stem cells (GSCs), which possess self-renewal capacity, multipotency, and the ability to regenerate tumor heterogeneity. GSCs contribute to key hallmarks of GBM pathobiology, including relentless progression, resistance to chemotherapy and radiotherapy, and inevitable recurrence. GSCs exhibit distinct molecular signatures, enhanced DNA repair, and metabolic adaptations that protect them against conventional treatments. Moreover, they reside within specialized niches—such as perivascular or hypoxic microenvironments—that sustain stemness, promote immunosuppression, and facilitate angiogenesis. Recent discoveries highlight signaling pathways like Notch, Wnt/β-catenin, Hedgehog, STAT3-PARN, and factors such as TFPI2 and HML-2 as critical regulators of GSC maintenance, plasticity, and immune evasion. These findings underscore the complexity of GSC biology and their pivotal role in driving GBM heterogeneity and therapeutic failure. Emerging therapeutic strategies aim to target GSCs through multiple avenues, including surface markers, immunotherapeutics (e.g., CAR T cells), metabolic vulnerabilities, and combination regimens. Advances in patient-derived organoids, single-cell omics, and 3D co-culture models enable more accurate representation of the tumor ecosystem and personalized therapeutic approaches. Ultimately, improved understanding of GSC-specific targets and the tumor microenvironment promises more effective interventions, paving the way toward better clinical outcomes for GBM patients.

## 1. Introduction

Glioblastoma (GBM) is the most aggressive and lethal primary brain tumor in adults, classified as IDH wild-type, grade IV astrocytoma by the World Health Organization [1]. Characterized by rapid proliferation, diffuse infiltration into surrounding brain tissue, extensive angiogenesis, and pronounced genomic instability, GBMs present significant clinical challenges [2]. A key factor contributing to the poor prognosis of GBM patients is the presence of GBM stem cells (GSCs) [3]. These cells exhibit stem cell-like properties, including self-renewal and the ability to generate diverse progeny that comprise the bulk of the tumor [4]. GSCs are thought to drive tumor initiation, progression, therapeutic resistance, and recurrence [5]. Understanding the role of GSCs is critical for developing novel therapeutic strategies aimed at improving patient outcomes [6].

## 2. Limitations of Current Therapeutic Strategies

The current standard of care for GBM involves maximal safe surgical resection followed by concurrent radiotherapy and chemotherapy with temozolomide, known as the Stupp protocol [7]. While this multimodal approach modestly extends survival, several limitations persist [8]. Due to the infiltrative growth pattern of glioblastoma, it is impossible to surgically remove all tumor cells without causing significant neurological deficits [9]. Residual microscopic disease at the tumor margins contributes to rapid recurrence [10]. Advanced imaging techniques like intraoperative MRI have improved resection, but cannot eliminate infiltrative cells [11].

GBM cells exhibit intrinsic and acquired resistance to temozolomide and radiotherapy [12]. The expression of O^6^-methylguanine-DNA methyltransferase (MGMT), which repairs temozolomide-induced DNA damage, is a well-known mechanism of chemoresistance [13]. Methylation of the MGMT promoter correlates with better responses to temozolomide, but this occurs in only a subset of patients [13]. Enhanced DNA damage repair pathways, activation of survival signaling, and hypoxia-induced radioresistance contribute to the limited efficacy of radiotherapy [14].

The supportive tumor microenvironment, including hypoxia, immunosuppressive conditions, and interactions with stromal cells, promotes tumor survival and resistance to therapies [15]. Hypoxic regions within the tumor activate hypoxia-inducible factors (HIFs), leading to angiogenesis, metabolic adaptations, and expression of genes that support tumor growth and resistance [16]. The immunosuppressive microenvironment impairs anti-tumor immune responses, further complicating treatment [17]. Additionally, many therapeutic agents fail to reach effective concentrations within the tumor due to the blood–brain barrier (BBB) and efflux transporters like P-glycoprotein [18]. Novel drug delivery systems, such as nanoparticles and convection-enhanced delivery, are being explored to overcome this barrier but have yet to demonstrate significant clinical benefit [19].

These obstacles highlight the critical need for innovative therapies that can overcome resistance mechanisms, effectively target tumor cells while sparing normal tissue, and address the complex biology of glioblastoma [20].

## 3. The Concept of Cancer Stem Cells in Glioblastoma

The cancer stem cell (CSC) theory proposes that within a heterogeneous tumor population, a subset of cells possesses stem cell-like properties, including self-renewal and the ability to generate diverse progeny that comprise the bulk of the tumor [21]. In glioblastoma, these cells are referred to as GBM stem cells (GSCs) and exhibit characteristics similar to normal neural stem cells (NSCs), such as the expression of stem cell markers (e.g., CD133, Nestin) and the capacity to differentiate into multiple neural lineages [22]. GSCs are capable of recapitulating the original tumor’s histopathology and genetic profile when implanted into animal models, confirming their role in tumor propagation [23]. The identification of GSCs has profound implications for understanding GBM biology and developing targeted therapies aimed at eradicating these root cells [24].

CSCs differ from the bulk of tumor cells in several key aspects [25]. They have the unique ability to self-renew indefinitely and generate heterogeneous tumor cell populations through differentiation [25]. This self-renewal is driven by the activation of specific signaling pathways and transcription factors that maintain stemness [26]. In contrast, bulk tumor cells have limited proliferative potential and are often more differentiated, contributing to the non-stem tumor cell population that comprises the majority of the tumor mass [27].

CSCs exhibit increased resistance to chemotherapy and radiotherapy compared to non-stem tumor cells [28]. This resistance is attributed to efficient DNA repair mechanisms, high expression of drug efflux pumps (e.g., ABC transporters), activation of anti-apoptotic pathways, and the ability to enter a quiescent state [29]. These properties enable CSCs to survive treatments that eliminate bulk tumor cells, leading to tumor recurrence [30]. CSCs are capable of initiating tumor growth when transplanted into immunodeficient animals, whereas bulk tumor cells often fail to form tumors under the same conditions [31]. Additionally, CSCs can exist in a quiescent or slow-cycling state, contributing to their resistance to therapies that target rapidly dividing cells [32]. Quiescent CSCs serve as a reservoir for tumor regrowth after treatment, highlighting the need for therapies that can target both proliferating and non-proliferating CSCs [33].

Understanding these differences is crucial for designing therapies that effectively target CSCs and prevent tumor recurrence [34]. Strategies aimed at eliminating CSCs or inducing their differentiation hold promise for improving treatment outcomes [35].

## 4. Characteristics of GBM Stem Cells (GSCs)

### 4.1. Identification and Isolation of GSCs

The identification and isolation of GSCs are critical for studying their biology and developing targeted therapies [36]. Surface markers have been widely used for this purpose [37]. CD133 (prominin-1) was one of the first markers identified. CD133-positive cells isolated from GBM specimens demonstrated enhanced tumorigenicity and stem-like properties [38]. However, relying on CD133 alone has limitations, as some studies have shown that CD133-negative cells can also exhibit stem cell characteristics and contribute to tumor growth [39]. This suggests that CD133 is not a universal marker for GSCs, necessitating alternative or additional markers [40].

Additional markers utilized include CD44, a cell surface glycoprotein involved in cell–cell interactions, migration, and adhesion [41]. CD44 is associated with the mesenchymal subtype of GBM and contributes to invasive behavior and therapy resistance [42]. Integrin α6 (CD49f) plays a role in cell adhesion and signaling. CD49f-positive cells possess stem-like properties and contribute to tumor initiation [43]. L1CAM (L1 Cell Adhesion Molecule), involved in cell migration and invasion, is associated with poor prognosis and increased tumor aggressiveness. Targeting L1CAM has been shown to suppress glioma growth in preclinical models [44]. A2B5, a ganglioside recognized by the A2B5 antibody, marks a subpopulation of GSCs with high tumorigenic potential [45].

Combining multiple markers improves the specificity and efficiency of GSC isolation [46]. For instance, cells co-expressing CD133 and integrin α6 have enhanced stemness and tumorigenicity [43]. However, heterogeneity among GSC populations and the overlap of markers with normal neural stem cells (NSCs) pose challenges [47]. Single-cell sequencing and proteomic analyses are being utilized to identify novel markers and better characterize GSC populations [48].

Functional assays are essential to confirm the stem-like properties of isolated cells [49]. The neurosphere formation assay is a widely used in vitro method where cells are cultured under non-adherent, serum-free conditions with growth factors like epidermal growth factor (EGF) and basic fibroblast growth factor (bFGF) [50]. GSCs form free-floating spherical clusters called neurospheres, indicative of self-renewal capacity [50]. The ability to generate neurospheres over multiple passages demonstrates long-term self-renewal [51]. The limiting dilution assay quantitatively assesses the frequency of stem cells within a population by determining the minimum number of cells required to form a neurosphere or initiate a tumor in vivo [52]. In vivo tumorigenicity assays involve transplanting putative GSCs into immunodeficient mice, where they can recapitulate the original tumor’s histopathology, infiltrative growth pattern, and heterogeneity, confirming their tumor-initiating capacity [53]. Additionally, differentiation assays assess the multipotency of GSCs by inducing them to differentiate into various neural cell lineages under specific culture conditions [54,55]. Assessing functional properties alongside surface marker expression provides a more comprehensive characterization of GSCs [34]. Table 1 details the common GSC markers and their significance.

### 4.2. Biological Properties of GSCs

GSCs possess robust self-renewal capacity, driven by intrinsic and extrinsic factors [56]. Intrinsic factors include transcription factors such as SOX2, OCT4, and NANOG, which are overexpressed in GSCs and regulate gene networks that promote stemness and inhibit differentiation [57]. Dysregulated signaling pathways contribute to the maintenance of GSC self-renewal [58]. Extrinsic factors involve microenvironmental cues; the tumor microenvironment provides signals that support GSC self-renewal, such as growth factors, cytokines, and extracellular matrix components [59]. Interactions with endothelial cells, pericytes, and immune cells influence GSC behavior [60].

GSCs exhibit distinct genetic and epigenetic profiles that differentiate them from non-stem tumor cells and normal NSCs [61]. Genetically, GSCs often harbor mutations characteristic of glioblastoma, such as alterations in TP53, PTEN, and EGFR genes [62]. Amplification of EGFR and expression of mutant forms like EGFRvIII enhance proliferative signaling in GSCs [63]. Epigenetically, GSCs display unique DNA methylation patterns and histone modifications that regulate gene expression related to stemness, differentiation, and therapy resistance [64]. For example, promoter hypermethylation of tumor suppressor genes like p16^INK4a^ leads to their silencing, facilitating uncontrolled proliferation [65]. Histone modifications influence chromatin structure and accessibility of transcription factors, affecting gene expression profiles associated with stemness and differentiation [66].

Recent studies have revealed that human endogenous retroviruses (HERVs), particularly HERV-K (HML-2), are pathologically expressed in malignant gliomas. Single-cell RNA sequencing identified GBM cellular populations with elevated HML-2 transcripts in neural progenitor-like cells (NPC-like) that drive cellular plasticity. Using CRISPR interference, it has been demonstrated that HML-2 critically maintains GBM stemness and tumorigenesis. HML-2 expression regulates embryonic stem cell programs in NPC-derived astroglia and alters their 3D cellular morphology by activating the nuclear transcription factor OCT4, which binds to an HML-2-specific long-terminal repeat (LTR5Hs). Targeting HML-2 with antiretroviral drugs reduces reverse transcriptase activity, tumor viability, and pluripotency, suggesting that HML-2 contributes fundamentally to the GSC niche and may serve as a unique therapeutic target [67].

### 4.3. Signaling Pathways Focusing on GSCs

The Notch, Hedgehog, and Wnt/β-catenin signaling pathways are crucial in regulating GSC self-renewal and survival [68]. Dysregulation of these pathways contributes to the maintenance of stemness and resistance to therapies [68]. In addition, the STAT3 pathway plays a critical role in GBM malignancy, including the maintenance of GSCs [69]. Recent research has identified poly(A)-specific ribonuclease (PARN), a key modulator of RNA metabolism, as a transcriptional target of STAT3 that activates EGFR-STAT3 signaling to support GSCs. PARN positively regulates self-renewal and proliferation of GSCs through its 3′–5′ exoribonuclease activity. By modulating EGFR expression via negative regulation of the EGFR-targeting miRNA miR-7, PARN creates a positive feedback loop to increase STAT3 activation. Targeting PARN in GSCs reduces tumor infiltration and prolongs survival in orthotopic brain tumor xenografts, suggesting that the STAT3-PARN regulatory network plays a pivotal role in tumor progression and represents a potential target for GBM therapeutics [70].

Another gene, TFPI2, has been identified as a major player in regulating GSC stemness and microglia immunosuppression. TFPI2 is amplified in a subset of GBM tumors, and its expression promotes GSC self-renewal through activating the JNK-STAT3 pathway. Additionally, GSC-secreted TFPI2 triggers the infiltration of microglia and causes them to become immunosuppressive in the tumor microenvironment. Inhibition of the signaling pathway impairs tumor growth, activates T-cells, and synergizes with therapy in GBM models. These findings provide new potential targets for treating aggressive tumors by disrupting the symbiotic interaction between GSCs and the immune microenvironment [71].

## 5. GSCs in Tumor Initiation and Progression

### 5.1. Role in Tumor Heterogeneity

GBMs are characterized by remarkable intra-tumoral heterogeneity, which significantly contributes to therapeutic resistance and disease progression [72]. This heterogeneity arises from the presence of diverse cellular subpopulations within the tumor, each exhibiting distinct genetic, epigenetic, and phenotypic profiles [48]. GSCs are central to the development and maintenance of this heterogeneity due to their capacity for self-renewal and differentiation into multiple cell lineages found within the tumor microenvironment [33].

Recent studies have further elucidated the complexities of GSC heterogeneity. Researchers created a targeted CRISPR library to screen 30 patient-derived GSC cultures for genetic dependencies specific to two distinct transcriptional subtypes: developmental and injury-response. The developmental subtype of GSCs is linked to neurodevelopmental processes and depends on transcriptional regulators crucial for neurodevelopment, whereas the injury-response subtype shows characteristics tied to tissue repair and immune response and relies on genes involved in integrin and focal adhesion signaling. These subtype-specific vulnerabilities reveal potential targets for precision therapies. For example, drugs targeting β1 integrin, FAK, MEK, and OLIG2 showed differential sensitivity depending on the GSC subtype [73]. Understanding these subtype-specific dependencies is crucial for developing effective, personalized treatments that address the inherent heterogeneity of glioblastoma. Table 2 illustrates the subtype specific vulnerabilities in GSCs.

Moreover, GSCs contribute to genetic diversity through the accumulation of mutations over time, leading to the emergence of subclones with distinct genetic profiles [10]. This clonal diversity within the tumor enhances its ability to adapt to therapeutic pressures and environmental changes, ultimately contributing to disease progression and recurrence [74].

### 5.2. Angiogenesis and the Vascular Niche

GSCs play a pivotal role in promoting angiogenesis and establishing the vascular niche within the tumor microenvironment [75]. GSCs secrete a variety of pro-angiogenic factors, including vascular endothelial growth factor (VEGF), fibroblast growth factor (FGF), and angiopoietins, which stimulate endothelial cell proliferation, migration, and new vessel formation [34]. VEGF, in particular, is a critical mediator of tumor vascularization and has been extensively investigated as a prime therapeutic target in GBM. However, targeting VEGF alone has shown limited efficacy, highlighting the complex nature of angiogenesis involving multiple pathways that warrant further investigation [48]. It has been shown that lymphatic endothelial-like cells (LECs), previously unrecognized in brain parenchyma, are present in GBMs and promote the growth of CCR7-positive GSCs through CCL21 secretion. Disruption of CCL21-CCR7 paracrine communication between LECs and GSCs inhibited GSC proliferation and growth. LEC-derived CCL21 induced KAT5-mediated acetylation of HMGCS1 on K273 in GSCs to enhance HMGCS1 protein stability, promoting cholesterol synthesis favorable for tumor growth. These findings highlight the complex role of endothelial cells in supporting GSC maintenance and offer potential therapeutic strategies targeting the vascular niche [76].

### 5.3. Immune Modulation

GSCs contribute to the immunosuppressive microenvironment of glioblastoma, hindering effective anti-tumor immune responses [77]. They secrete immunosuppressive cytokines such as transforming growth factor-beta (TGF-β), interleukin-10 (IL-10), and prostaglandin E2 (PGE2), which inhibit the activation and proliferation of effector immune cells, including T cells and natural killer (NK) cells [78]. GSCs also express immune checkpoint molecules like programmed death-ligand 1 (PD-L1), interacting with programmed death-1 (PD-1) receptors on T cells, leading to T cell exhaustion and apoptosis [79]. This interaction reduces the cytotoxic activity of T cells against tumor cells [79]. GSCs modulate the phenotype and function of antigen-presenting cells (APCs) such as dendritic cells and macrophages, inducing immunosuppression and impairing the initiation of effective immune responses [80]. The mechanisms of GSC-mediated immunosuppression are summarized in Table 3.

## 6. GSCs and Therapy Resistance

### 6.1. Mechanisms of Chemotherapy Resistance

GSCs exhibit resistance to chemotherapy through several mechanisms, including overexpression of drug efflux transporters and enhanced DNA repair capabilities [81]. The overexpression of ATP-binding cassette (ABC) transporters, such as ABCG2 and P-glycoprotein (ABCB1), actively pumps chemotherapeutic agents out of the cells, reducing intracellular drug accumulation and efficacy [81]. The expression of ABC transporters is regulated by signaling pathways associated with stemness, such as Hedgehog and Wnt/β-catenin pathways [82]. Activation of these pathways in GSCs leads to the upregulation of ABC transporters, enhancing drug efflux capacity [82].

### 6.2. Metabolic Adaptations

GSCs exhibit metabolic adaptations that support their survival and contribute to therapeutic resistance [83]. Rewired amino acid metabolism can lead to an altered tumor immune microenvironment and enhanced tumor growth [84]. Lysine catabolism is reprogrammed in GSCs, with increased extracellular lysine uptake mediated by upregulation of the lysine transporter SLC7A2. Accumulation of crotonyl-coenzyme A (crotonyl-CoA), a bioactive intermediate metabolite of lysine catabolism, and subsequent crotonylation of histone H4 lysine (Kcr) occur in GSCs due to enhanced expression of the crotonyl-CoA-producing enzyme GCDH and reduced expression of the crotonyl-CoA hydratase ECHS1. Depletion of GCDH abolishes the induction of crotonyl-CoA and Kcr, leading to upregulation of type I IFN signaling genes and senescent markers. These findings suggest that GSCs reprogram lysine catabolism to support tumor growth and an immunosuppressive microenvironment [85].

**Figure 1:**
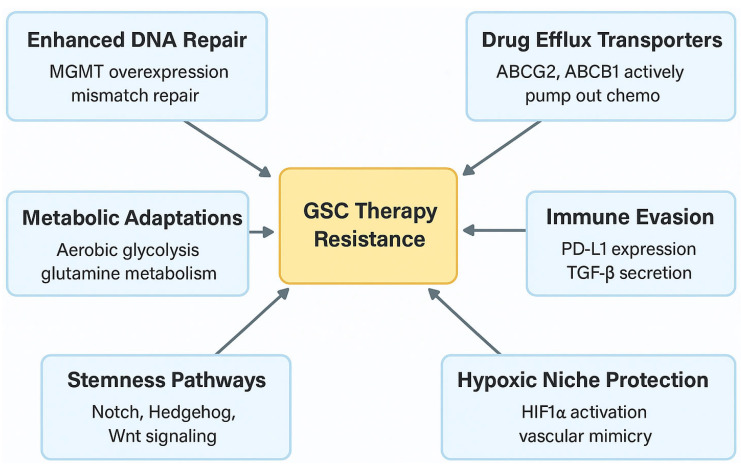
Key mechanisms of GSC therapy resistance.

Therapeutically, the combined use of lysine restriction along with a MYC inhibitor or anti-PD-1 immunotherapy synergistically impairs Kcr and GSC growth both in vitro and in vivo without additional observed toxicities. This indicates that GSCs reprogram lysine catabolism to induce type I IFN signaling and affect cell fate [85]. Figure 1 shows the key mechanisms of GSC therapy resistance.

## 7. Therapeutic Strategies Targeting GSCs

### 7.1. Targeting Surface Markers

Surface markers uniquely expressed or overexpressed on GSCs provide accessible targets for therapeutic interventions [86]. Monoclonal antibodies and antibody-drug conjugates (ADCs) have been developed to recognize and eliminate GSCs based on these markers [87]. CD133 is one of the most studied GSC markers [38]. Monoclonal antibodies targeting CD133 have been investigated to selectively eliminate GSCs [39]. However, challenges arise due to CD133’s expression on normal hematopoietic stem cells and other progenitor cells, raising concerns about off-target effects and potential toxicity [88]. Antibody-drug conjugates targeting other GSC surface markers like CD44 and EphA2 have shown promise in preclinical studies [89].

### 7.2. Immunotherapeutic Approaches

Harnessing the immune system to target GSCs offers a promising avenue for therapy [90]. Immunotherapeutic strategies include vaccines, adoptive cell therapies, and immune checkpoint inhibitors [90]. Chimeric antigen receptor (CAR) T-cell therapy involves genetically modifying T cells to express receptors that recognize tumor-specific antigens, enabling them to target and kill tumor cells [91]. CAR T cells targeting GSC markers such as interleukin-13 receptor alpha 2 (IL13Rα2), HER2, EGFRvIII, and the ganglioside GD2 have been developed [92]. A phase 1 clinical trial (NCT04003649) is ongoing to evaluate the efficacy of IL13Rα2 CAR T cells when given alone or in combination with nivolumab and ipilimumab [93].

CAR T cells targeting cell surface GRP78 (csGRP78) have been shown to efficiently kill GBM tumor cells and GSCs both in vitro and in vivo, ultimately suppressing xenograft tumor growth without causing significant tissue injuries. The expression of csGRP78 is increased on the surface of GBM cells and GSCs in response to endoplasmic reticulum stress, while it is restricted to the cytoplasm and nucleus in normal cells. Targeting csGRP78 represents a valuable strategy for effective immunotherapy against GBM [94]. Alongside CAR T cells, Chimeric antigen receptor natural killer (CAR-NK) cell therapy is another emerging adoptive cell therapy approach being explored for glioblastoma. CAR-NK cells may offer potential advantages, such as a lower risk of graft-versus-host disease and the possibility for ’off-the-shelf’ allogeneic use, and investigations are underway to assess their efficacy against GSCs and the broader tumor [3,94].

Additionally, a phase 1 clinical trial evaluated a next-generation CAR-T therapy, CARv3-TEAM-E T cells, in treating recurrent GBM. This novel therapy combines CAR-T with T-cell-engaging antibody molecules (TEAMs) to address tumor heterogeneity by targeting mixed tumor cell populations. The therapy showed rapid tumor regression in patients, highlighting the potential of cell therapy for solid tumors like GBM [95]. There are several studies underway investigating safety and feasibility against multiple common targets. For example, while a phase I study (NCT05353530) is designed to assess the safety and efficacy of IL-8 receptor-modified CD70 CAR T cell therapy in CD70+ glioblastoma, another phase I trial (NCT04214392) is studying the side effect and dose of CAR T cells with a chlorotoxin tumor targeting domain in patients with MPP2+ GBM [96,97]. Moreover, a phase I (NCT03726515) is exploring CAR T cell therapy in combination with ICI therapy in GBM [98].

## 8. Challenges in Targeting GSCs

### 8.1. Marker Heterogeneity and Specificity

One of the primary obstacles in targeting GSCs is the absence of universal and exclusive markers that distinguish them from normal neural stem cells (NSCs) and other tumor cells [99]. Markers such as CD133, CD44, and Nestin are commonly used to identify GSCs but are also expressed in normal tissues [100]. Moreover, GSCs exhibit significant heterogeneity in marker expression, with subpopulations lacking these markers still possessing stem-like properties [101]. This heterogeneity complicates the development of therapies that can effectively target all GSC populations [47]. Reliance on a single marker may result in incomplete eradication of GSCs and eventual tumor recurrence [102].

### 8.2. Tumor Microenvironment Influence

The tumor microenvironment provides protective niches that shield GSCs from therapeutic interventions [103]. Hypoxic regions within the tumor promote GSC maintenance and resistance to therapy [104]. The vascular niche, supported by aberrant angiogenesis, supplies nutrients and survival signals to GSCs [60]. These protective niches hinder the penetration and efficacy of therapeutic agents [105]. Additionally, the microenvironment can modulate GSC phenotypes, promoting quiescence or activating survival pathways in response to stress [106]. Strategies to disrupt these niches or modulate the microenvironment may enhance the accessibility and effectiveness of GSC-targeted therapies [107].

### 8.3. Therapeutic Resistance and Adaptation

GSCs can activate redundant or compensatory signaling pathways to maintain stemness and survival when targeted therapies inhibit a specific pathway [108]. This redundancy poses a significant challenge to monotherapies targeting a single pathway [61]. Combination therapies targeting multiple pathways simultaneously may be necessary to prevent compensation and achieve more effective GSC eradication [109].

## 9. Future Perspectives

### 9.1. Advancements in GSC Research Models

Traditional two-dimensional (2D) cell culture models have been invaluable for studying cancer biology, but fall short in replicating the complex architecture and microenvironment of tumors in vivo [110]. Three-dimensional (3D) co-culture models and bioprinting technologies have emerged as advanced models that more accurately mimic the structural and functional characteristics of GBMs [111]. For instance, the interaction between mesenchymal stem cells (MSCs) and glioblastoma, although potentially of high importance, is not fully understood [111]. The role of MSCs in the GBM microenvironment is complex and actively investigated, with studies exploring both pro- and anti-tumorigenic effects mediated by MSC-derived paracrine factors. These factors can influence GSC behavior, potentially inhibiting proliferation while enhancing migration, and modulate key signaling pathways like Wnt and TGF-β [3,109]. Advanced models are crucial for dissecting these interactions. Using 3D co-culture systems, researchers have studied how MSCs might acquire characteristics of cancer-associated fibroblasts (CAFs) when exposed to GBM-conditioned medium, leading to the formation of MSCCAFs [109,110,111]. Co-culturing MSCCAFs with patient-derived GBM cells in scaffold-based 3D bioprinted models further allows for the investigation of therapeutic responses within a more relevant context. Such models enhance our understanding of the dynamic cell–cell interactions within the tumor microenvironment, providing valuable insights for identifying novel therapeutic targets aimed at disrupting the supportive niche [53,111].

Using 3D co-culture systems, researchers can study how MSCs can acquire characteristics of cancer-associated fibroblasts (CAFs) when cultured with conditioned medium from GBM cultures, leading to the formation of MSCCAFs [111]. Co-culturing MSCCAFs with patient-derived GBM in a scaffold 3D bioprinted model allows for the study of responses to current GBM therapy [111]. Such models enhance our understanding of cell–cell interactions between the tumor microenvironment and cancer cells, providing insights into novel therapeutic targets [111].

Patient-derived xenografts (PDXs) and organoids also serve as valuable tools for studying GSCs and testing therapeutic strategies [112]. By using GSCs isolated from patient tumors to establish PDX models, researchers can observe how these cells contribute to tumor growth and response to therapies within a living organism [112]. Organoids maintain the genetic mutations, epigenetic alterations, and phenotypic diversity of the original tumor, providing a more physiologically relevant model for research [113].

### 9.2. Single-Cell Omics and Personalized Medicine

The advent of single-cell omics technologies has revolutionized the understanding of tumor heterogeneity and the molecular intricacies of GSCs [114]. Single-cell RNA sequencing (scRNA-seq) enables the analysis of gene expression profiles at the individual cell level, uncovering the diversity of cellular states within a tumor [115]. Applying scRNA-seq to GBMs has revealed that GSCs are not a homogeneous population but consist of multiple subpopulations with distinct molecular signatures and functional properties [116]. Personalized medicine aims to customize treatments based on the unique molecular characteristics of a patient’s tumor [117]. By leveraging single-cell omics data, clinicians can develop tailored therapeutic strategies that target the specific vulnerabilities of a patient’s GSCs [118].

### 9.3. Combination Therapies

Combating GBM effectively may require combination therapies that integrate GSC-targeted treatments with standard modalities such as surgery, chemotherapy, and radiotherapy [119]. The rationale behind this approach is to eliminate both the bulk tumor cells and the GSCs responsible for recurrence and resistance [120]. For instance, combining Notch pathway inhibitors with temozolomide and radiotherapy can enhance the sensitivity of GSCs to these treatments [121]. Integrating immunotherapies targeting GSC-specific antigens with conventional treatments may enhance anti-tumor immune responses [122]. When designing combination therapies, it is crucial to consider potential synergistic effects as well as additive toxicities [123]. Optimizing dosing regimens and timing is essential to maximize therapeutic efficacy while minimizing adverse effects [124].

## 10. Discussion and Future Directions

The intricate biology of glioblastoma stem cells (GSCs) positions them at the nexus of glioblastoma’s most challenging characteristics: its profound heterogeneity, formidable therapeutic resistance, and inevitable recurrence [5,6,125]. As explored in this review, GSCs leverage their capacities for self-renewal and differentiation not only to initiate and propagate tumors but also to actively sculpt the tumor microenvironment, fostering angiogenesis and orchestrating immune evasion [34,77,126]. Significant strides have illuminated key molecular underpinnings of GSC function, identifying critical signaling pathways (e.g., Notch, Wnt, Hedgehog, STAT3-PARN), metabolic dependencies (e.g., lysine catabolism), immunomodulatory factors (e.g., TFPI2, PD-L1), and distinct transcriptional subtypes (developmental vs. injury-response) that govern their maintenance and plasticity [67,68,70,71,73,85,127]. These discoveries pave the way for innovative therapeutic strategies, ranging from targeted inhibitors and metabolic interventions to sophisticated immunotherapies like CAR T-cell constructs directed against GSC-associated antigens (e.g., IL13Rα2, csGRP78) [85,92,94,95].

However, a fundamental challenge pervades GSC research and complicates the direct translation of these findings: the lack of a universally accepted, definitive method for identifying and isolating these elusive cells. While the use of cell surface markers such as CD133, CD44, A2B5, L1CAM, and Integrin α6 has been instrumental in enriching for populations with stem-like properties [38,41,42,43,44,45], considerable evidence highlights the limitations of this approach. Marker expression is often heterogeneous and dynamic, influenced by culture conditions, microenvironmental cues, and therapeutic pressures [39,47,101]. Crucially, studies have demonstrated that marker-negative populations can retain significant tumorigenic potential and exhibit functional stem cell characteristics in vivo [39], questioning the exclusivity and stability of these markers as definitive GSC identifiers.

This definitional ambiguity necessitates careful interpretation of studies investigating GSC biology and therapeutic responses. Much of our current understanding, including insights into signaling pathways, metabolic adaptations, and immune interactions discussed herein (e.g., [67,70,71,77,85]), often relies on GSC populations isolated or defined using specific marker profiles (e.g., CD133+) or functional assays like neurosphere formation [50,53]. While invaluable, these methods may capture distinct GSC subpopulations or cellular states rather than the entirety of the functional GSC compartment. Furthermore, emerging evidence from single-cell analyses suggests that GSC identity might be more fluid than previously appreciated, representing a spectrum of cellular states characterized by specific transcriptional programs (e.g., developmental vs. injury-response) rather than a rigidly defined entity [48,73,116]. Cells may transition between states, acquiring stem-like functionality under specific conditions, further complicating static, marker-based definitions [3,115].

Consequently, advancing the field requires a paradigm shift towards a more integrated, multi-faceted approach to characterizing GSCs. Relying solely on one or two surface markers is insufficient. Future investigations should rigorously combine multi-parameter marker analysis with robust functional validation, employing assays like limiting dilution analysis and in vivo tumorigenicity assays in diverse preclinical models [52,53]. Critically, the integration of single-cell multi-omics (transcriptomics, epigenomics, proteomics) is essential to dissect the inherent heterogeneity, plasticity, and state transitions within the functional GSC pool [114,115]. Utilizing advanced preclinical models, such as patient-derived organoids and xenografts that better recapitulate the tumor architecture, microenvironment, and cellular heterogeneity [111,113], will be vital for studying GSCs in a relevant context and validating therapeutic strategies aimed at functionally defined stem-like populations.

A more nuanced, functionally validated understanding of GSC identity, states, and heterogeneity is paramount for developing effective therapeutics. Strategies targeting surface markers, while conceptually appealing, must contend with expression variability and potential off-target effects [88,101]. Immunotherapeutic approaches like CAR T-cells show immense promise [92,94,95], but their success hinges on identifying targets consistently expressed on the functionally relevant GSC populations and overcoming the immunosuppressive milieu GSCs help create [77,79]. Targeting metabolic vulnerabilities [85] or specific signaling dependencies revealed by subtype analysis [73] offers alternative routes, potentially less reliant on unstable surface markers. Ultimately, given GSC adaptability and the complexity of GBM, combination therapies targeting multiple vulnerabilities simultaneously—integrating GSC-specific agents with standard-of-care (chemotherapy, radiotherapy) and strategies to modulate the tumor microenvironment—appear essential for overcoming resistance and preventing recurrence ([109,119], Table 4).

## 11. Conclusions

Glioblastoma Stem Cells represent a critical driver of GBM malignancy, underpinning tumor heterogeneity, immune evasion, and therapeutic failure. While significant progress has been made in deciphering the molecular pathways and interactions governing GSC behavior, the inherent plasticity and definitional challenges associated with these cells remain major hurdles. Overcoming these requires a move towards integrated research methodologies combining multi-omics, functional assays, and advanced preclinical models to capture the dynamic nature of GSCs. Future therapeutic success will likely depend on developing personalized, multi-modal strategies that not only target specific GSC vulnerabilities—be they surface antigens, signaling pathways, or metabolic dependencies—but also account for their heterogeneity and adaptive resistance mechanisms, potentially by co-targeting the supportive tumor microenvironment. Continued innovation, rigorous validation in relevant models, and collaborative efforts are essential to translate our growing understanding of GSC biology into tangible clinical benefits for patients battling this devastating disease.

## Figures and Tables

**Table 1:** Common and emerging glioma stem cell (GSC) markers and their functional significance.

Marker	Expression/Localization	Key Functional Role	Relevance in GSC Biology	Potential for Targeted Therapy	Additional Comments
CD133	Cell-surface glycoprotein (also known as Prominin-1)	Maintenance of stem-like phenotype; Self-renewal	Widely used to isolate GSCs; Expression correlates with poor prognosis	Potential immunotherapy target (e.g., vaccines, antibodies); Strategies under investigation	- Expression can be lost under certain culture or therapeutic conditions - Not entirely exclusive to GSCs, but still a dominant marker
CD44	Cell-surface adhesion molecule	Cell adhesion and migration; Contributes to mesenchymal/invasive properties	Enriched in mesenchymal GSC subtypes; Facilitates brain infiltration	Blockade strategies (e.g., antibodies, small-molecule inhibitors) explored	- Multiple isoforms can exert varied effects on GSC properties - Linked to therapy resistance
A2B5	Cell-surface ganglioside marker	Identifies glial precursor-like cells	Widely co-expressed with other stem markers (e.g., CD133); Helps refine GSC populations	Possible immunotherapeutic target in combination with other markers	- Commonly used in combination panels to increase specificity for GSC isolation
L1CAM	Cell-surface adhesion molecule	Promotes cell motility and adhesion; Enhances invasiveness	Crucial for GSC maintenance and survival; Associated with radiation resistance	Monoclonal antibodies under development	- Regulates invasive potential - Elevated expression tied to poor patient outcomes
Integrin α6	Cell-surface receptor for laminin	Mediates cell-ECM attachment; Promotes survival and invasion	High levels correlate with stemness; Facilitates basement membrane infiltration	Integrin inhibitors in clinical or preclinical evaluation	- Targeting α6 can reduce GSC viability - Often combined with radiation or chemotherapy for synergy
Nestin	Intracellular intermediate filament protein	Structural support in progenitor cells	Neural stem/progenitor cell marker; Reflects high proliferative capacity	Not directly targeted; Primarily used for GSC identification	- Expression often correlates with tumor aggressiveness and poor prognosis
SOX2	Intracellular transcription factor	Maintains pluripotency and self-renewal	Essential for GSC proliferation; Drives stem-like gene programs	Various small-molecule inhibitors under early investigation	- Central regulator of neural development pathways - Overexpression linked to aggressive disease
OLIG2	Intracellular transcription factor	Regulates oligodendrocyte lineage commitment; Contributes to neuronal specification	Critical for GSC proliferation; Associated with radioresistance	Potential gene therapy or epigenetic modulation	- Frequently upregulated in specific GBM subtypes - High OLIG2 often predicts worse outcomes
BMI1	Intracellular polycomb group protein	Chromatin remodeling; Governs self-renewal	Promotes GSC survival; Linked to therapy resistance and aggressiveness	Epigenetic inhibitors targeting BMI1 are being tested	- Overexpression correlates with poor clinical outcomes - Maintains stemness under environmental stress
ALDH1A3	Cytoplasmic enzyme (aldehyde dehydrogenase)	Detoxification; Retinoic acid metabolism	Enriched in tumor-initiating GSC subpopulations; Associated with chemo- and radioresistance	ALDH inhibitors show promise in preclinical models	- Useful for isolating more aggressive GSC populations - Predictive of therapy resistance
PDLIM1	Intracellular scaffold protein containing PDZ and LIM domains; also known as CLP36	Regulates proliferation, apoptosis, and tumorigenesis; Maintains/expands GSC subpopulations; Confers chemoresistance (via PI3K-AKT pathway)	Specifically enriched in GSCs within GBM; Drives poor prognosis and therapy resistance	Novel target for inhibiting GSC-mediated tumor growth and resistance	- Newly identified GSC marker in GBM; Knockdown reduces GSC ratios and tumorigenic potential - Likely modulates cytoskeletal reorganization and downstream signaling (e.g., PI3K-AKT)

**Table 2:** Subtype-specific vulnerabilities in glioblastoma stem cells (GSCs).

GSC Subtype	Developmental	Injury-Response
Key Transcriptional Programs and Features	- Linked to neurodevelopmental processes - High expression of neurodevelopmental TFs (e.g., OLIG2, SOX2) - Often enriched for pathways driving progenitor-like phenotypes	- Associated with tissue repair and immune/inflammatory signaling - Activated integrin/focal adhesion pathways - Often co-express markers tied to stress response (e.g., integrin α6)
Unique Dependencies/Vulnerabilities	- OLIG2 expression critical for proliferation and GSC maintenance - Neurodevelopmental signaling hubs (e.g., Hedgehog, Notch)	- Integrin-FAK axis for adhesion, migration, and survival - Upregulated MAPK/MEK signaling - Stress-related survival pathways (e.g., NF-κB)
Targeted Pathways/Genes	- OLIG2 - SOX2 - Hedgehog/Notch signaling	- β1/α6 integrins - FAK (focal adhesion kinase) - MEK (in MAPK pathway)
Representative Experimental Drugs or Approaches	- OLIG2 inhibitors (gene therapy or small-molecule approaches) - Hedgehog/Notch pathway inhibitors (e.g., vismodegib for Hedgehog; γ-secretase inhibitors for Notch)	- FAK inhibitors (e.g., defactinib) - Integrin-blocking antibodies (e.g., volociximab targeting α5β1 or cilengitide targeting αvβ3/αvβ5) - MEK inhibitors (e.g., trametinib, cobimetinib)
Rationale/Mechanism	- Blocking OLIG2 disrupts core developmental programs critical for GSC self-renewal. - Inhibiting developmental pathways (Hedgehog, Notch) impairs stemness and forces differentiation, potentially reducing tumor propagation.	- Disrupting FAK or integrin signaling decreases GSC migration, adhesion, and survival within the injured/tumor microenvironment. - MEK inhibition blocks downstream survival and proliferation signals. - Targeting these nodes selectively impairs ’injury-response’ GSCs reliant on these pathways.

**Table 3:** Mechanisms of GSC-mediated immunosuppression.

Mechanism	Important Molecules	GSC-Driven Effects	Potential Interventions
Secretion of immunosuppressive cytokines	- TGF-β - IL-10 - PGE2	- Suppresses T cell, NK cell function - Reduces pro-inflammatory cytokine production - Induces regulatory T cell (Treg) expansion	- TGF-β inhibitors (e.g., galunisertib) - IL-10 blocking antibodies - COX-2 inhibitors (to reduce PGE2)
Immune checkpoint molecule expression	- PD-L1 - PD-1 (on T cells)	- Inhibits T cell activity via PD-1/PD-L1 axis - Promotes T cell exhaustion - Leads to apoptosis of effector T cells	- Anti-PD-1 (e.g., nivolumab) - Anti-PD-L1 (e.g., atezolizumab) - Combination with other immunotherapies
GSC-modulated APC dysfunction	- Dendritic cells - Tumor-associated macrophages (TAMs) and microglia - GSC-secreted TFPI2 (JNK-STAT3 activation)	- Induces tolerogenic DCs with impaired antigen presentation - Polarizes macrophages/microglia toward immunosuppressive phenotypes (M2-type) - Promotes Treg cells	- DC-based vaccines with matured DCs - Macrophage reprogramming (e.g., CSF-1R inhibitors) - TFPI2 pathway inhibitors
Rewiring of amino acid metabolism	- Lysine transporters (SLC7A2) - Crotonyl-CoA-producing enzyme (GCDH) - Crotonyl-CoA hydratase (ECHS1)	- Alters immune cell infiltration and activation - Promotes an immunosuppressive microenvironment - Dampens type I IFN signaling	- Targeted inhibition of lysine transporters - Crotonylation inhibitors - Dietary lysine restriction + MYC/PD-1 blockade
Enhanced extracellular stress response	- Cell surface GRP78 (csGRP78) - ER stress pathways	- Upregulated under ER stress - Creates an escape mechanism from immune surveillance - Potentially reduces cytotoxic T cell recognition	- CAR T cells targeting csGRP78 - ER stress modulators

**Table 4:** Combination strategies against GSCs.

Combination Strategy	Mechanism of Action	Synergy and Key Findings	Evidence (Preclinical/Clinical)
GSC-Targeted Inhibitors + SOC (TMZ/Radiotherapy)	- Inhibition of GSC survival pathways (e.g., Notch, Hedgehog, Wnt/β-catenin) - DNA damage from TMZ/radiation	- Targeted inhibitors sensitize GSCs to DNA-damaging agents - Reduced ability of GSCs to repair DNA lesions under pathway inhibition	Preclinical and early-phase trials
Small-Molecule EGFR Inhibitors (e.g., Erlotinib) + SOC	- Blockade of EGFR signaling, reducing proliferation - TMZ/radiation damage increases reliance on the EGFR pathway	- Enhanced apoptosis in GSCs that rely on EGFR survival signaling - Prolonged survival in xenograft models	Preclinical studies
Immune Checkpoint Inhibitors + GSC-Targeted Therapy	- Immune re-activation (anti-PD-1, anti-CTLA-4) - Direct blockade of GSC-maintaining pathways (e.g., STAT3, TFPI2)	- Encourages T-cell-mediated elimination of GSCs - Decrease in immunosuppressive factors secreted by GSCs	Ongoing clinical trials
CAR T Cells + Standard-of-Care	- CAR T cells specifically target GSC surface markers (e.g., IL13Ra2, EGFRvIII, csGRP78) - TMZ or radiation to reduce tumor bulk	- Dual targeting: bulk tumor reduction by SOC plus immunologic targeting of therapy-resistant GSCs - Potential for durable responses	Phase 1/2 clinical trials
Metabolic Inhibitors (e.g., Lysine Restriction) + Immune Tx	- Interference with GSC-specific metabolic pathways (e.g., lysine catabolism) - Enhanced T-cell response (anti-PD-1, adoptive T cells)	- Disruption of GSC metabolic reprogramming - Synergistic effect on immune activation and GSC depletion	Preclinical models
Angiogenesis Inhibitors (Bevacizumab) + GSC-Directed Agents	- VEGF pathway inhibition disrupts the vascular niche supporting GSCs - GSC-targeted agents (e.g., integrin α6 inhibitors) impair adhesion and survival	- Decreased blood supply to the GSC niche - Reduced ability of GSCs to invade and self-renew	Preclinical and clinical settings
Epigenetic Modulators (HDAC/BMI1 Inhibitors) + SOC	- Reversal of GSC-associated epigenetic changes - TMZ/radiotherapy exert cytotoxic effects	- Epigenetic sensitization of GSCs to DNA-damaging therapies - Inhibition of self-renewal pathways (BMI1, etc.)	Preclinical
Multi-Targeted Approach: GSC-Targeted Vaccine + Checkpoint Inhibitors + SOC	- Vaccine primes the immune system against GSC-specific antigens - Checkpoint blockers sustain T-cell activity - SOC reduces tumor mass	- Immunological ’double hit’: vaccine-activated T cells plus checkpoint blockade - Reduced immune evasion by GSCs - Lower tumor burden	Early clinical trials

## Data Availability

No new data was generated in this manuscript.
